# What Makes the Life of Stressed Plants a Little Easier? Defense Mechanisms against Adverse Conditions

**DOI:** 10.3390/plants12051040

**Published:** 2023-02-24

**Authors:** Ewa Muszyńska, Kinga Dziurka, Mateusz Labudda

**Affiliations:** 1 Department of Botany, Institute of Biology, Warsaw University of Life Sciences-SGGW, Nowoursynowska 159, 02-776 Warsaw, Poland; 2 Department of Biotechnology, The Franciszek Górski Institute of Plant Physiology, Polish Academy of Sciences, Niezapominajek 21, 30-239 Kraków, Poland; 3 Department of Biochemistry and Microbiology, Institute of Biology, Warsaw University of Life Sciences-SGGW, Nowoursynowska 159, 02-776 Warsaw, Poland

## 1. Introduction

Plants experience a wide array of external factors, some of which negatively affect their metabolism, growth, and development. Such unfavorable circumstances, collectively called environmental stress, require plants’ mechanisms to overcome these pressures and reach homeostasis if they want to survive and reproduce in the stressful environments in which they find themselves. The current Special Issue, entitled ‘What makes the life of stressed plants a little easier? Defense mechanisms against adverse conditions’ is a compilation of fifteen papers (original and review) related to structural, metabolomics, proteomics, and genomics changes occurring under various stress conditions. In this way, it covers the latest advances in our multifaceted understanding of responses and adaptation strategies of plants, mainly to water scarcity and elements’ imbalance, but also to other stressors such as non-optimal temperature or fungal disease.

## 2. Drought Stress

The problem of drought and maintaining efficient crop production in a changing climate is a challenge for people around the world. Research is therefore focused on understanding the mechanisms of plant defense reactions against water deficit, as well as on the search for markers of drought resistance and substances modulating plant response to a stress factor. Expanding the knowledge in this field by integrating different approaches not only prevents crop losses, but also accelerates the process of breeding drought-resistant varieties. Wahab et al. [1] reviewed the effects of drought stress on earlier germination and flowering, the morphology of leaves, stems, and roots, as well as crop yields. The authors discussed changes in physiological and biochemical parameters, such as leaf relative water content (RWC), photosynthesis efficiency, stomatal conductivity, respiration, membrane stability, accumulation of osmolytes (proline, soluble sugars, glycine betaine) and antioxidant enzymes, and non-enzymatic reactive oxygen species (ROS) scavengers (carotenoids, phenolics). Finally, they showed the possibility of inducing tolerance to drought using exogenous phytohormones and changes in the content of endogenous phytohormones under drought stress (auxins, cytokinins, gibberellins, abscisic acid (ABA), ethylene, jasmonates).

Going deeper into the details, Hassan et al. [2] studied the application of nano-silicon (nSi) as a regulator improving the biochemical and physiological parameters of drought-sensitive ‘Kalamata’ olives grown in semi-arid climates with insufficient rainfall. The ‘Kalamata’ olive reacted to drought stress with oxidative stress manifested by an increase in H_2_O_2_, malondialdehyde (MDA), electrolyte leakage, and fruit drop. The use of nSi in the form of foliar spray at a concentration of 150–200mg/L^−3^ under moderate drought (90% of irrigation water requirements) resulted in improved yield, total chlorophyll content, and RWC, reduced fruit drop, and simultaneously lowered the content of proline, soluble sugars, H_2_O_2_, MDA, ABA, and electrolyte leakage. The authors postulated that nSi increases plant tolerance to drought by reducing the production of ROS and thus alleviating oxidative stress.

Drought stress usually does not occur alone but is accompanied by temperature stress: cold or heat. Hence, the influence of several stressors on the condition and physiology of plants is being studied more and more often. Terletskaya et al. [3] studied the effect of water deficit and cold on the morphology, physiology, and biochemistry of the medicinal plant *Rhodiola semenowii* Boriss. The authors noticed non-specific reactions, such as a decrease in photosynthesis efficiency or the accumulation of tocopherol in shoots, beta-sitosterol in roots, and squalene in both shoots and roots, regardless of the type of stress. At the same time, they observed specific changes in the anatomy of *R. semenowii* under studied stress factors. The root, shoot, and leaf cells were flattened, with reduced turgor under drought stress, and more hydrated, rounded, and densely packed in cold than control.

Drought is often accompanied by osmotic stress. Paunescu et al. [4] applied a polyethylene glycol (PEG) solution in hydroponic cultivation to mimic drought stress to screen winter wheat varieties of various origins for the activity of antioxidant enzymes. Increased peroxidase activity under the 25% PEG treatment might suggest their resistance to drought in most Romanian varieties. On the other hand, varieties considered to be drought-tolerant showed low ascorbate peroxidase activity compared to the control. The authors concluded that none of the tested biochemical markers can be a clear indicator of drought tolerance due to the lack of correlation between the yield index and the ratio of antioxidant enzyme content between PEG and control.

## 3. Chemical Elements’ Stress

The effect of elevated levels of metallic elements resulting from strong environmental contamination of anthropogenic origin is one of the most frequently studied issues of both basic and applied sciences. In the experiment by Niedziela et al. [5], triticale (*×Triticosecale* Wittm. ex A. Camus) lines differing in aluminum (Al) tolerance were compared regarding their ability to root regrowth and proteomic changes that may help in recovery after Al stress. The authors demonstrated that the roots of the Al-tolerant triticale line could recover without modification of proteome profiles. On the contrary, Al-sensitive genotypes maintained the proteome alteration caused by unfavorable environments. The highest upregulation was detected for proteins involved in protein folding (i.e., protein disulfide-isomerase), stress-related response (such as glutathione *S*-transferase, oxalate oxidase, and 1-Cys peroxiredoxin), and flavonoid metabolism (flavone O-methyltransferase 1), while proteins involved in cell division (tubulin) and metabolic pathways associated with amino acid metabolism and methylation control (*S*-adenosyl-L-homocysteine hydrolase), as well as ascorbic acid biosynthesis (phosphomannomutase), were downregulated. In turn, Chrysargyris et al. [6] noted that the physiological markers of plant response to metal-induced stress are often beneficial bioactive secondary metabolites, mainly antioxidants employed in the food, pharmaceutical, and cosmetic industries. Thus, they hypothesized that the treatment of the medicinal plant *Pelargonium graveolens* with different levels of copper (Cu) ions could stimulate the synthesis of phenolic compounds with high ability to scavenge of ROS. *P. graveolens* plants; these proved to be surprisingly tolerant to Cu stress, and in general, the applied concentrations of Cu up to 100 μM did not have a negative influence on biomass production and the tested physiological parameters, such as stomatal resistance, chlorophyll *a* fluorescence, and photosynthetic pigment contents. The observed tolerance of *P. graveolens* was attributed to both the detoxification of most of the Cu ions in roots, the restriction of their translocation to shoots, and an efficient antioxidant system based on flavonoids and other, undetected classes of phenolics with strong ROS neutralization properties. Taken together, these results showed that the leaves of *P. graveolens* can be safely exploited as an herbal raw material which can be used for the extraction of bioactive compounds, the biosynthesis of which might be stimulated by plant exposure to Cu. On the other hand, the review paper of Labudda et al. [7] showed the significance of basic sciences in the development of applied sciences. According to the authors, the understanding of plant adaptation and acclimation mechanisms in response to harsh conditions is necessary to relieve the pressure of environmental changes and ensure global food security for an increasing population, as well as to restore areas degraded by human activity. Therefore, special attention was paid to the natural strategies of metallophytes and hyperaccumulators that exhibit microevolutionary adaptation to high concentrations of metals in the soil. The authors highlighted the possibility of the practical application of metal-tolerant species in various phytoremediation techniques, as well as indicating similarities between metal tolerance mechanisms and those activated during salinity and drought. Finally, they discussed in detail the latest scientific achievements in priming methods regarding the use of phytohormones, nanoparticles, reactive chemical species, and (non-)ionizing radiations, which may effectively induce a defense response to subsequently occurring stress, and provide plant acclimation and improved resistance to heavy metals, salinity, and water deficit.

The chemical ion imbalance in soil can also result from the excessive accumulation of Na^+^, K^+^, Cl^−^, NO_3_^−^, and SO_4_^2−^ in the soil solution, and this disturbance is responsible for salinity stress. The study conducted by Purmale et al. [8] aimed to compare salinity tolerance and ion accumulation ability between the *Armeria maritima* subsp. *elongata* accession from geographically isolated, salt-affected habitats. Interestingly, the authors used both in vitro shoot culture and soil-cultivated plants for examination. It was found that the increasing concentration of NaCl under in vitro treatment negatively influenced shoot multiplication and biomass production, probably due to the lack of a natural mechanical barrier (i.e., roots with Casparian strips) to ion translocation to shoots. On the contrary, the growth of *A. maritima* under greenhouse cultivation was not significantly affected by increasing salinity, and Na^+^ or K^+^ treatment had a similar effect on plants. However, there were differences in osmotic adjustment (inorganic ions vs. organic osmolytes) between these two cations. Tolerance to salinity in *A. maritima* was achieved by the deposition of salt crystals on the leaf surface and flower stalks, as well as by the storage of toxic ions in the oldest leaves, and therefore they were discarded by the plants.

## 4. Varia

Delving into the molecular basis of plant responses to biotic and abiotic stress factors, Kesawat et al. [9] studied the proline-rich extensin-like receptor kinases (PERKs) gene family in *Triticum aestivum* L. PERKs genes are known to be involved in plant development and stress responses. The authors identified thirty seven genes in wheat from this family (TaPERKs) and confirmed their increased expression under stress conditions. Many of the TaPERK genes were up-regulated during the Septoria tritici blotch, powdery mildew, or stripe rust infections. A few of them were also expressed under hot and cold stress. The TaPERK gene family does not appear to directly participate in drought stress. In addition, different genes of the TaPERK family have been shown to respond to various stress factors. Their molecular role in plant responses was also assigned to the SKP1-CUL1-F-box (SCF) type E3 ubiquitin ligases, which participate in multiple specific protein degradation, including those that permit survival under stress conditions. SCF complexes use F-box (FBX) proteins as renewable substrate adaptors to levy proteins for ubiquitylation. The F-BOX STRESS INDUCED (FBS) subfamily of plant FBX proteins has an abnormal structure, nevertheless, with an F-box domain placed in the center and extra conserved regions at the N- and C-termini. Sepulveda-Garcia et al. [10] showed two WD40 repeat-like proteins in Arabidopsis that are highly conserved in plants and interact with FBS proteins, which were named FBS INTERACTING PROTEINs (FBIPs). FBIPs react only with the N-terminus of FBS proteins, and this interaction takes place in the nucleus. The authors concluded that FBS proteins may act in stress-responsive nuclear events, and described two WD40 repeat-like proteins as new tools to study how the abnormal SCF complex, SCFFBS, functions through the interaction events of N-terminal FBX proteins.

Continuing with stress responses at the cellular level, the review paper of Li et al. [11] indicated that calcium ions are one of the important regulators of plant reactions to various abiotic stresses, such as water deficit and excess, salt stress, light stress, heavy metals, non-optimal temperatures, and mechanical stimuli. In addition, the authors presented the crosstalk between Ca^2+^ and other signaling molecules (ROS, ABA, nitric oxide, inositol 1,4,5-trisphosphate, cyclic ADP-ribose, cyclic guanosine 30,50-monophosphate) in plants under environmental challenges. In turn, from an organismal point of view, plants can react to stressors through the formation of the secondary abscission zone (SAZ), which allows them to eliminate a damaged organ or its fragment from parent individuals. Dziurka et al. [12] elucidated the mode of action of methyl jasmonate (JA-Me) in inducing the formation of SAZ, as well as analyzing changes in the content of endogenous phytohormones above and below the SAZ in the stems of *Bryophyllum calycinum*. It was found that changes in the metabolism of auxin and jasmonic acid-related compounds accompanied the formation of SAZ under the influence of JA-Me. At the same time, the authors did not observe any modification of the indole-3-acetic acid (IAA) content (which can also induce SAZ), suggesting that the mode of action of JA-Me in inducing SAZ is different than in the case of IAA.

A promising way to relieve plant stress is the involvement of microbiota, which—working with the host plant—improves the condition of the plant and often enables it to survive. A perfect example of this *Brachypodium distachyon*, in which the composition of the leaf microbiome changed under the influence of acclimatization to cold temperatures [13]. The relative abundance of *Streptomyces* sp. M2, which releases antibiotics and siderophores known to inhibit the growth of pathogens, increased by over 200-fold. At the same time, the amount of *Pseudomonas syringe*, an ice nucleation active pathogen that can incite frost injury to crops, drastically decreased. *Streptomyces* spp. produced heat shock proteins and cryoprotectants which could protect the host plant during acclimatization to cold temperatures. On the other hand, many microorganisms can be pathogenic and bring about a serious threat to crop productivity. Regarding this issue, Rabby et al. [14] screened a new bioactive secondary metabolites against the most damaging fungal disease of wheat. The authors noted that two marine secondary metabolites—bonactin and feigrisolide C, isolated from the marine bacteria *Streptomyces* spp.—significantly inhibited the growth of *Magnaporthe oryzae* hyphae in vitro. In further analyses, the authors found that bonactin and feigrisolide C decreased the mycelial growth of this pathogen in a dose-dependent manner. Bonactin reduced mycelium growth more effectively than feigrisolide C. The authors noted that, to be able to recommend these molecules as fungicides for wheat blight control, the further study of additional *M. oryzae* isolates is needed to determine their exact mode of action and disease control effectiveness under various field conditions.

Next, a literature review, which is more species-specific in its scope, concerns the response to abiotic stress of cultivated beet (*Beta vulgaris* L.) [15]; this review shows selected aspects of molecular, biochemical, physiological, and structural responses to heavy metals, alkalines, non-optimal temperatures, and UV stresses. Here, attention was also paid to changes in the expression of selected genes that seem to play important roles in response to the abiotic stresses of beet plants. The use of this kind of approach and description regarding this topic, as the authors also noted, may be useful in future works on the topic of breeding.

## 5. Conclusions and Future Perspectives

The excellent articles published in this Special Issue summarize and broaden the latest achievements in the field of stress biology. The shared knowledge gathered here, showing what can make the lives of stressed plants a little easier, can provide clues for the development of new strategies (or for the improvement of those already in existence) to combat the global problems faced by the modern world, including drought, metal pollution, salinity, and climate changes. Such a multi-perspective view of plant complexity may bring about a promising solution for the restoration of degraded lands, as well as the conduction of sustainable agriculture and improved quality and productivity of plants growing under diverse, even stressful conditions. On the other hand, more research is needed on at least two different stresses acting simultaneously to fill the gaps in our knowledge concerning plant responses to external stimuli that usually occur collectively in nature. Another scientific challenge is also to discover both crosstalk connections between particular metabolic pathways and signaling cascades responsible for efficient and rapid plant adaptation and acclimation to changing environments.

As Guest Editors, we are grateful to all the authors for choosing to publish their vulnerable research in our Special Issue. We also greatly appreciate the work of the reviewers who took the time to review all of the submitted manuscripts. We believe that, together, we have contributed to a better cognition and understanding of compound plant reactions to multiple stresses and the determination of future perspectives and trends.

## Data Availability

Not applicable.
